# Error in References Section

**DOI:** 10.1001/jamanetworkopen.2020.36103

**Published:** 2021-01-14

In the Original Investigation titled “Risk Factors Associated With In-Hospital Mortality in a US National Sample of Patients With COVID-19,”^1^ published December 10, 2020, there was an error in the References section. The References section incorrectly listed the article by Arshad et al^2^ as reference 26. The correct reference is Boulware et al.^3^ This article has been corrected.^1^
